# Biodigestion System Made of Polyethylene and Polystyrene Insulator for Dog Farm (on the Example of the Republic of Chile)

**DOI:** 10.3390/life12122039

**Published:** 2022-12-06

**Authors:** Cristian Vivallos Soto, Francisco Ruiz Bertín, Carolina Robles Calderón, Maxim Viktorovich Larionov, Priscila Jackeline Arias Ordóñez, Ivette Cevallos Baque

**Affiliations:** 1 Peoples Friendship University of Russia (RUDN University), 6 Miklukho-Maklaya Street, 117198 Moscow, Russia; 2 Biopecuaria Limited, Plot 4 A, Las Bandurrias, Pirque, Santiago 9480000, Chile; 3 Faculty of Ecology and Environmental Protection, Russian State Social University (RSSU), 4 Wilhelm Peak Street, Building 1, 129226 Moscow, Russia; 4 Institute of Industry Management, State University of Management (SUM), 99 Ryazanskij Prospect Street, 109542 Moscow, Russia; 5 World-Class Scientific Center “Agrotechnologies for the Future” (CAAT), Russian State Agrarian University—Moscow Timiryazev Agricultural Academy, 49 Timiryazevskaya Street, 127550 Moscow, Russia; 6 Federal State Budgetary Educational Institution of Higher Education “State University of Land Use Planning” (SULUP), 15 Kazakov Street, 105064 Moscow, Russia

**Keywords:** organic waste, canine feces, equine manure, manure processing, biodigester, biogas, bioenergy, psychrophilic conditions, chemical analyzes, polyethylene, polystyrene, sanitary and hygienic safety, environmental safety, soil and ecosystem protection

## Abstract

Anaerobic digestion is a system that can have a high environmental impact through the use of different wastes to obtain biogas and its consequent use for the generation of renewable energy. The objective of this study was to implement a polyethylene biodigester, using polystyrene for thermal insulation in a dog kennel, using canine feces collected in the same place during a period of 5 months to obtain biogas and energy. The results indicated that biogas production started on day 30 and stopped during the winter period with low temperatures; therefore, from day 54 onwards, equine manure was added to continue producing biogas. Although biogas was obtained, the biodigester did not function optimally, due to the fact that the materials used in its construction did not provide efficient insulation from the low external temperatures; the low C/N ratio of the canine feces, which led to a reduction in the processing of the methanogenic bacteria; and the low amount of feces collected for use. In general, the use of a biodigester can provide a tool for the biological processing and management of organic waste, yielding a cumulative source of renewable energy and ensuring environmental safety.

## 1. Introduction

The modern world is faced with various environmental problems. Moreover, this has been observed in various regions of the world. Currently, there are many threats to the environment [1,2,3,4,5,6]. Modern nature management practices have caused serious disturbances in the environment. Nature management has led to the weakening of ecosystems in various territories. This has been observed in the conditions of various industrial complexes [7,8,9,10], transport systems [11,12,13], and settlements [14,15,16], as well as natural [6,17,18,19] and natural-economic complexes [20,21,22,23]. Waste is of particular importance in relation to the deterioration of the quality of the environment, and in the oppression and weakening of ecosystems and human health.

In connection with the development of agriculture and related trends in the national economy (energetic nature management, urban planning, urbanization, agglomeration, and the development of transport systems and various industries), new spaces are being developed. Agricultural production can have many negative effects on the created and surrounding natural ecosystems, on soil biota, and on the resource quality of the soil itself as an ecologically valuable bio-inert system.

For example, in plant growing, there are a number of urgent problems with direct economic and environmental effects. There is a clear deficit in the influx of organic matter into soils in the form of plant litter in agricultural land and urban green areas. Thus, as a result, these soils do not receive the required amount of substances required for the normal development of plants and, in general, for the optimal functioning of natural and cultural phytocenosis. This leads to the violation of the biogeochemical cycles of nature-like ecosystems. At the same time, the introduction of foreign substances from organic waste exacerbates the ecological situation. The accumulation and unsystematic storage of organic waste makes a significant contribution to the deterioration of the ecological state and stability of soils and ecosystems. Dehumification—the destruction and loss of ecological-physical and ecological-biochemical potential—can occur in soils. The economic quality of cultural ecosystems is declining. In natural ecosystems, processes of structure simplification, the violation of biocenotic relationships, irreversible changes in dynamic characteristics, and general degradation have been observed.

The problem of waste generation in crop production is quite obvious. In particular, stubble burning is still very widespread in different regions of the world. This action leads to many problematic environmental situations. Some of these processes also lead to dehumification and significant volumes of greenhouse gases entering the atmosphere. Burning plant residues can also lead to fires and other negative manifestations of this type of unreasonable management.

In some cases, tallow from agricultural crops can be used to a limited extent as an acceptable biofuel [23,24,25,26]. Studies have indicated that plant wastes can be used to build bioprocessing facilities to obtain biofuels and other useful products [27,28,29,30,31]. It is important to control the process at all stages. Much attention should be paid to the reagents and conditions used.

One of the most serious threats to the environment is animal waste [32,33,34,35], and the demand for livestock products is not decreasing; on the contrary, the demand for animal husbandry has tended to increase [36]. Waste generation from animal husbandry is an urgent environmental problem. Measures to protect the environment are required. This is also necessary in relation to the consequences of environmental pollution related to waste from animal husbandry. Currently, the formation of animal waste is a serious environmental problem. Measures are needed to protect the environment. This is also necessary in relation to the consequences of environmental pollution due to animal waste.

In many cases, animal waste can pose real threats to ecosystems, human health, and environmental quality [36,37,38,39,40,41,42,43]. Undoubtedly, animal husbandry waste should be more widely used in the national economy [42,43]. This has been mentioned many times in a number of articles [44,45,46,47,48,49,50]. This is especially expedient when there is a trend towards an increase in the demand for livestock products and, accordingly, against the background of an increase in livestock production [36,51,52,53]. Currently, a steady increase in livestock production and the accompanying increase in waste is a global trend.

Chile is a country that is incorporating non-conventional renewable energy sources (NCRE) into its energy matrix through incentives and subsidies for their use and development, and incorporating legislative changes that seek to reduce energy dependence on fossil fuels and their associated externalities [54]. Nevertheless, there are still many problems in the field of approbation and implementation of developments for efficient waste management, collection, processing and disposal. The situation with the violation of the ecological balance in the exploited landscapes and the high pathogenic danger for the population and for the domestic animals themselves remains. This is celebrated in vast areas of South America and in the Republic of Chile itself. An effective approach among the opportunities for ensuring environmental safety in the system of organic waste management is their widespread collection and subsequent processing. Biodigestion of organic waste and other unused biological objects in this case may be of some importance. Thus, it is possible to eliminate the ecological and pathogenic danger to human and animal health, to natural vegetation, to cultivated plants and soils.

The anerobic digestion of waste is a clean, simple, and low-cost form of non-conventional renewable energy (NCRE), in relation to other technologies of this type [55]. In addition, it provides the ability to use a great variety of animal manure, agricultural residues, and waste from the food industry, generating various agricultural and socioeconomic benefits through the reduction of odors, the inactivation of pathogens, and the generation of a renewable and clean fuel for use in multiple applications [56,57,58,59,60,61,62]. In Europe, livestock waste is considered to have high biogas potential; e.g., Sweden has the potential to generate an average of 3–6 TWh per year [63,64]. At the same time, the production of biogas is accompanied by the formation of a number of products for practical use.

Biogas production can be generated domestically or in small production systems, and its performance can be associated with various factors, such as the local conditions of the site, where the environmental temperature (T°) is relevant [65,66,67], and the substrate used.

The use of biogas is most efficient as an environmentally friendly fuel and in biofertilizers, as well as for effective waste management, the decontamination of toxins, and effective pathogen control [68,69,70,71,72]. The process of obtaining biogas is based on the biological degradation of animal feces through anerobic digestion [73,74,75,76]. Waste from animal husbandry can be used in various ways [77,78,79,80,81,82,83,84]. In general, the direction of application considered here, in the system of organic waste management, has great prospects.

Among the forms of animal waste that can be used as a substrate to generate biogas through anerobic digestion is canine feces. Although canine feces has a low potential for biogas generation and a high retention time [72], it is capable of producing a sufficient quantity for small-scale use and its biogas production can be enhanced by mixing it with other plant and animal wastes [85].

To analyze the production of biogas using dog feces under local conditions, the objective of this study was to develop and implement a polyethylene biodigester in a dog kennel to optimally treat manure and generate biogas to be used in the premises of the same kennel, evaluating the functionality of polyethylene (PE) in isolating the ambient temperature to allow the survival of methanogenic bacteria. For the reactor, another type of plastic such as polyvinyl chloride (PVC) can be used, and four tubular digesters can be installed, functioning as four phases of the same biochemical process, to ensure a high level of biogas production [86,87].

## 2. Materials and Methods

In this study, canine feces obtained from the Commercial Bigpatagons Ltd.a. Kennel were used, located in the Pirque commune, Cordillera province, located to the south east of the Metropolitan Region of Chile (33°43′08.5″ S, 70°30′43″ W). This region is the smallest in the country; however, it contains 40.33% of the national population [88].

In the field, a pre-feasibility study was developed, in which various aspects, parameters, characteristics, and basic and current data were evaluated in order to understand what type of biodigestion plant was required for the treatment of this waste for biogas production, and adjacent factors were also considered. Likewise, based on previous bibliographic data, a specific sketch of the biochemical parameters, dimension, designs, function, treatments, and production of the system was made.

Subsequently, a feasibility study was developed for the physicochemical and biological analysis of the biomass, the treatment of the manure and the determination of the potential of biogas that would be generated, as well as to determine the system’s design, its size, build, and function, thus developing a particular conceptual model of the campus.

### 2.1. Physicochemical and Biological Analysis

The physicochemical and biological analysis of the biomass (canine manure) was carried out by Agrolab Laboratory, to obtain the percentage of dry matter (DM), organic matter or volatile mass (VM, which is the volume of the previous parameter) and the load of volumetric organic compounds (VOC). The availability of biomass was 30 kg, with a collection percentage of 100% (Table 1).

### 2.2. Development of the Biodigester’s Dimensions

The parameters calculated for the development of the biodigester were as follows: DM; VM; dilution (the necessary amount of water to add to the manure to form a solution with 10% dry matter); specific volume (considering 2.2 L and the availability of biomass); and hydraulic retention time (HRT), which was estimated based on the average annual T° of Pirque, which was 14.2 °C [89]. Therefore, it was determined that the operating range of the biodigester would correspond mostly to psychrophilic methanogenic bacteria, and to a lesser extent to mesophilic bacteria.

When the biodigester operates at low temperatures, which are indeed suitable for psychrophilic bacteria, there are lower ammonium concentrations (increases of which would cause toxicity towards bacteria); therefore, failures would not be caused during anaerobiosis [90]. In any case, in the case reactors with small dimensions and those that operate at low temperatures, their diverse population of microorganisms must be studied in order to optimize digestion [91].

HRT is closely linked to the type of substrate and its T°; the lower the T°, the higher the HRT; the greater the volume of the digester for biomass, then the lower the volume of CH_4_. Thus, the HRT was estimated to be 30 days. The theoretical solid retention time (SRT) was determined based on the HRT, which, for a simple continuous-flow biodigester, without solid retention, was equal to HRT. The volume of the loading and unloading tank, i.e., the volume of the biodigester at its base, was calculated [92,93]. Furthermore, the VOC was determined, considering the kg of daily VM divided by the total volume (in m^3^) of the biodigester per day (Table 2).

The base of the biodigester (without the geomembrane) had a volume capacity of 2.44 L (at maximum 3.0 L) of a mixture of manure and water, which was converted into cubic meters for the biodigester, which had a capacity of 2.5 m^3^ of capacity (maximum 3 m^3^) [92,93]. Considering that the higher the organic load of the biodigester (and therefore the larger its dimensions) the greater the volumetric generation of biogas, we used mesophilic and thermophilic operating temperatures [94].

### 2.3. Biodigester Design

Regarding technical innovation and according to the results of the feasibility study, a semi-continuous biodigester was developed—a prefabricated Chinese model (or covered lagoon, with a fixed dome) [95] made of polyethylene material. Structured PE or PVC biodigesters are simpler to operate (without requiring an expert) and are cheaper (by up to 70%) than those built out of materials such as steel [96]. Studies on the biochemistry of methanogenic psychrophilic bacteria and the implementation of low-cost PE for reactors, have demonstrated their feasibility in obtaining high levels of biogas even with a PE thicknesses of caliber 8, which may help to avoid the influence of low external temperatures [97]. It is necessary to consider the fact that if the system is implemented with high temperatures (by natural or artificial means), this will stimulate the development of thermophilic methanogenic bacteria in the reactor, which can more effectively increase the generation of biogas, compared to reactors using mesophilic and psychrophilic bacteria [98] and although high-temperature anaerobic digestion studies have often corroborated this outcome, there are some studies showing different results [99].

Innovations were applied to the design of the model. The first was the use of a loading and unloading tank in the thick base of the model, reducing construction costs compared to those made of cement or concrete while maintaining a similar quality to preserve the biochemical process prevailing inside the biodigester, according to the temperature required by the psychrophilic (and mesophilic) bacteria, and (unlike other waste treatments) generating biogas [92].

Our second innovation was the implementation of structural insulated panel (SIP)-type expanded polystyrene panels, with oriented strand board (OSB)—type wood with the use of magnesium oxide specifically adapted to the system to efficiently insulate the biomass present inside, which needed a high temperature to survive. In addition to ensuring this, the biogas produced by the system is optimally used, since it is not used to heat this organic matter.

As a third innovation, a manual mixer was manufactured to be used in the mixing-loading tank, since prior to biodigestion, mixing and homogenization must be carried out, helping the 10% solution of feces and water (that is, 10% feces and 90% water) to reach a state that is suitable for biodigestion. The DM of 23% indicates that this canine manure was classified as solid and had a medium-high content of solids [98]. At first glance, this appeared compact, in that its solid content was the product of feeding mostly with pellets, which have a high amount of fiber and types of fibrous proteins, with little vegetable content and little moisture. Therefore, a more efficient apparatus was required to homogenize the feces. This apparatus was manufactured with ordinary metal, in the form of propellers, inside the tank, with seventeen blades with lengths of 10, 20, and 30 cm and with widths of 0.5 and 1 cm, holding them with a mixing lever on the outside of the tank, which was separated from the inside by the lid of the latter. This lever was L-shaped, 40 cm long, 0.5 thick, and 1 cm wide, and it rotated the propellers 360°. Mixing was carried out for a maximum time of 5 min.

### 2.4. Infrastructure

The biodigestion system was assembled on the premises of the Commercial Bigpatagons Ltd.a. (Santiago, Chile) kennel. The dimensions of the system were as follows: 4.5 m long, 1.5 m high, and 1.4 m in total diameter (biodigester, loading tank, discharge tank, and pipes). The biodigester in its base material had a volume of 3 m^3^, and adding the geomembranes it had a total capacity of 6 m^3^ and 6.3 m^2^, giving 3150 L of useful volume. The horizontal-type reactor was built underground to avoid heat loss, with 10 cm thick inlet and outlet pipes made of polyethylene and PVC, respectively, surrounded by 10 cm thick SIP panels. Prior to the biodigestion process, a proximal analysis of the fecal material to be inoculated was performed, determining the pH, the amount of organic matter, the amount of dry matter, total nitrogen, C/N ratio, and the amount of minerals.

### 2.5. Functioning

After the analysis of the substrate, we proceeded to the collection and entry of the loading tank with 4 kg of canine feces and 28 L of water (32 L total, the first filling), which were mixed naturally and with a metal mixer with 5 blades. After obtaining a suitable mixture, a metal filter was used to prevent impurities from entering directly through a pipe to the biodigester load. The duration of the trial was 5 months, with a retention time of 30 days. From day 54, equine feces was mixed with dog feces due to lack of the latter when winter began. The loading and unloading procedure was carried out periodically, five, four and three times a week, randomly.

The quantities of manure (in kg) and water (in L) entered each time were calculated by means of an electronic weight, the temperature of the biodigester was calculated by means of a basic thermometer, and the environmental temperature was based on the information from the Chilean meteorological service at its meteorological station in the area of Pirque.

**Figure 1:**
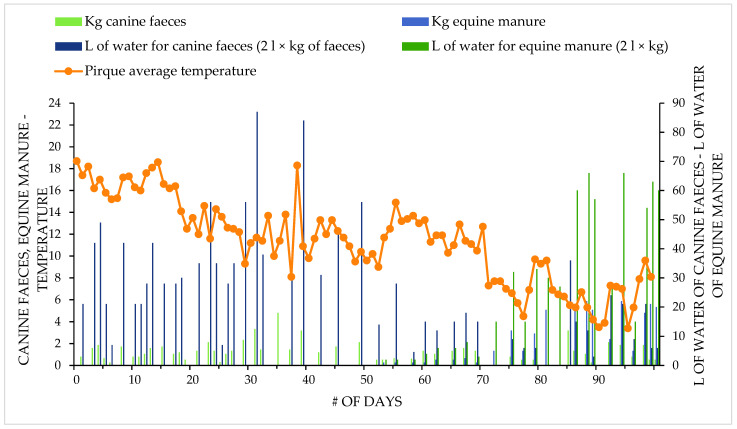
Features of the considered process related to kg of feces.

**Figure 2:**
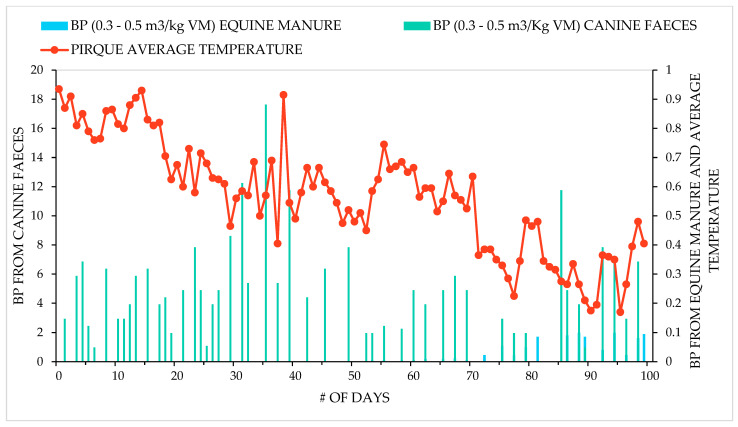
Features of the considered process related to BP.

The theoretical biogas production (BP) is expressed in m^3^/ day/ BP and this was estimated according to the dimensions of the biodigester base; 1 m^3^/volume is equal to 1 m^3^/day/minimum BP, which is more accurate than other methods (i.e., using kg/day/MV or by measuring chemical oxygen demand and biological oxygen demand), since once the system is working, the specific BP is governed directly by the volume of the biodigester. The volume of the biodigester (with a thick base without a geomembrane) for this dog farm was 3 m^3^, so there would be 3 m^3^/day/BP. In any case, the exact BP calculation was obtained after the biodigester was used to treat the manure (Figure 1 and Figure 2).

## 3. Results and Discussion

The use of polyethylene in the construction of the biodigester fulfilled the objective of generating biogas, although unlike other studies in which biogas production could be observed from the first day of implementation [100], biogas was obtained after day 30 onwards in the digester geomembrane, but in periods with low temperatures, no generation was observed. In this project, there was no electricity generation based on the use of biogas because its theoretical quantity was low, at 0.58 m^3^/Kg MV of canine feces and 2.07 m^3^/Kg/MV of equine manure, and also due to the observation of the expansion of the geomembrane by biogas The generation of electricity is feasible in plants that generate over 10 m^3^/day of gas, since a minimum of 1 m^3^ of biogas is required to produce 1 kWh of electricity.

### The Biodigester Did Not Work Completely Due to the following Factors

1. Environmental temperature: During the experiment, in the Pirque area, the average temperature was 14.2 °C and the lowest temperature recorded was 0.2 °C. This affected the activity of the bacteria, which reach an optimal biodigestion level at mesophilic temperatures from 25° to 45°.

It was not completely useful, in terms of thermal insulation, to use polyethylene for the base of the biodigester since, although its walls were thick, they poorly maintained and the internal temperatures required to protect bacteria from the influence of external temperatures; they only separated the organic matter of the biodigester from the outside, requiring the use of an additional structure or the attachment of devices to maintain optimal temperatures. The polyethylene structure may work better if solar panels are added to the outside of the structure to deliver energy to the internal radiator in the tank, maintaining a warm temperature for the biomass [101].

The incorporation of thermal insulators, such as the assembly of a wooden greenhouse with plastic or glass [102] or a plastic greenhouse [103], could represent a better solution than the use of SIP-type expanded polystyrene panels or glass wool, with OSB-type wood, because such a system would allow the sun’s rays to penetrate the structure and achieve the required environmental temperature so that the external temperature does not affect the biodigestion process. If this system was installed in a PE greenhouse that obtained good results during the daylight hours, it would also be necessary to ensure that the structure was hermetically sealed in order to prevent the influence of cold weather during the night (maintaining more thermal inertia and less air exchange) [97]. In addition, a heat control unit could be used during colder seasons to increase biogas production [104].

2. Mixer: The mixer used did not fully meet our expectations, since in the loading tank, after mixing, some uncrushed or unmixed pieces of feces were observed. These entered the biodigester, and at the outlet in the discharge tank, pieces of undigested feces were still found as a result of obtaining an ineffective initial fecal mixture in the mixing-discharge tank. In larger biodigestion systems, mechanical mixers with or without choppers or grinders (the latter being electrically powered) are used; in small projects, manual mixers are often used, although there is no setting limiting the choice of mixer [98,105].

A possible solution to this problem is the incorporation of a more efficient mixer that could include a chopper or even a basic grinder, obtaining the energy for its operation from the biogas generated by the biodigester itself, or failing that, a manual mixer/grinder, similar to the one used in the present study, but with a greater number of blades to ensure optimal chopping, mixing, and homogenization of the solution. The choice of the mixer should take into account the type of manure used, since, as was observed in this study, dog feces has a very solid consistency due to its high dry matter content. An example of this is slaughterhouse waste, in which the confiscation (organs and tissues of the animal that are not going to be consumed) has a solid structure and for which a specific grinding process is required prior to the mixing tank when this type of waste is used in biodigesters [106]. As a dog feces grinder, the use of a manual grinding apparatus for organic products (such as meat) would have been useful, which due to its structure also functions as a mixer [107].

3. The Chinese model reactor: A different biodigester design could be tested, since semi-continuous biodigesters work optimally with contents of 8–12% DM [105] and dog feces has a dry matter content of 23% DM; however, other studies have not determined that different designs from the semi-continuous ones should be used for feces with medium-high or medium DM percentages [72,106]. The plastic batch reactor model has demonstrated a high biogas yield, although this was achieved with small tanks with a capacity of two liters [108].

4. Carbon–nitrogen ratio (C/N). According to Table 1, dog feces has a very low C/N ratio of 12.4:1, which caused less processing of methanogenic bacteria [72,109]. According to [110], a C/N ratio between 25 and 30 is optimal for digestion; methanogenic bacteria consume 30 times more carbon than nitrogen, so optimal ratios between 10:1 and 30:1 or between 20:1 and 30:1 have been proposed [105]. Studies have even determined that for dog feces, a ratio higher than 16:1 is considered a low C/N ratio [103]. Therefore, to compensate for the low C/N ratio of the feces, the biodigester must be loaded with sawdust or shavings, which have a high contribution of carbon, of which the C/N ratio is very high (ranging from 200 to 300) [111]; thus, there would be enough carbon that the bacteria could use to consume the nitrogen contained in high levels in the feces more rapidly [72]. However, on the other hand, sawdust and shavings contain a high percentage of lignin, which is not easy to digest by means of anerobic digestion, generating a barrier that could make it impossible to decompose the other components of the mixture [106]. In order to solve this problem, the lignin content could be reduced quickly through the maceration of the sawdust/shavings or a compost could be prepared [105]. However, in other experiments with canine feces in which researchers have added sawdust and shavings, the opposite findings have been obtained, helping the dry matter to be digested in an optimal way [111]. These elements are mostly preferred for co-digestion since systems related to dog feces tend to occupy patio areas.

As a second option for raw materials that provide a high C/N ratio, one could add the remains of vegetables such as wheat and corn cereal straw, including grass, cut grass, and dry leaves (which, in addition, all have a low lignin content) [105] or sheep, goat, or bovine feces, which have an inoculum rich in methanogenic bacteria that will guarantee the production of biogas [112]. Likewise, according to the theoretical comparison with bovine manure, the mixture of canine waste with vegetable biomass generates levels of biogas similar to that of bovines [113]; researchers have even identified potential vegetables that could be used to produce biodiesel that may enable co-digestion with bovine dung, generating a relevant increase in biogas production, such as *Jatropha*, a de-oiled cake [114], and others waste products with their oils extracted which can used for biodiesel and for biogas [115]. However, there are combinations that can decrease the anerobic process and generate less biogas, such as the addition of chicken manure, which contains high levels of ammonium, causing an imbalance in the digestion steps and thus leading to the potential accumulation of volatile fatty acids [98].

5. The shaker: By not including an apparatus in the digester tank to agitate the substrate, there was insufficient homogenization of the mixture and no biomass mixing, nor was there the prevention of crusting, sedimentation, and foaming, which in turn prevented the maintenance of an adequate bacterial density and available sites of action for the bacteria to be able to act. The incompletely digested waste prevented the biochemical process from being effective, thus resulting in low biogas production. However, the implementation of a circulation pump inside the reactor would guarantee an effective mixture of the biomass, and this could be an alternative [100].

A suitable agitator can be implemented in a digester to adjust its internal biomass, depending on the reactor model. Studies have not specified that this device cannot be implemented for process efficiency [116]. Rural, small, and low-tech biodigesters contain agitators, which do not work continuously, and which are operated through cranks. Furthermore, daily loading of the reactor can be carried out for the purpose of agitating the mixture [117], but the canine digester examined in this study was not loaded daily.

Even for a reactor with dimensions of 0.5 m^3^, an agitator is needed (residential-style tanks can be made of steel). An agitator that guarantees efficiency can be made of AISI 316L stainless steel, which works well in humid environments and is resistant to temperature differences and corrosion [107].

## 4. Conclusions

The use of polyethylene as the base material for a biodigester, on the one hand, made the design and construction of the system easier, in addition to producing biogas. However, the physical characteristics of this plastic material did not isolate the biomass in an effective way from the low temperatures the external environment, affecting the biochemical processes of the digester bacteria, and with low biogas generation. The polystyrene panels used as peripheral thermal insulators were not effective to fulfill their purpose in the biodigester.

A manual mixer with blades or a mechanical-type mixer must have an appropriate design in order to properly homogenize the canine feces with water, due to the physical characteristics of this type of solid manure; otherwise, it will only work partially.

Since canine fecal samples have a very low C/N ratio, sawdust or shavings should be added (from the same dog beds) to augment carbon levels and improve anerobic digestion. In addition, the amount of canine feces that is usually available in rural and urban areas is not sufficient to produce biogas in quantities capable of generating electricity. This situation has generated little interest in relation to the development of biodigesters, which indicates the need for further studies to design engines that work with lower biogas yields. Co-digestion with equine manure did improve biogas generation or electric generation. However, the co-digestion of dog feces with other urban pruning waste or the organic fraction of urban solid waste should be strongly considered, since there was an evident increase up to 27% in biogas production, with 78.6% to 79.2% methane richness observed over time. Methanogenic bacteria from dog feces can further metabolize cellulose in plant mass and the addition of microorganisms from those substrates seems to help produce more biogas.

Therefore, it is necessary not only to dispose of organic waste from animals, it is advisable to use this waste for household needs. An ecological effect is also achieved through this approach in terms of ensuring environmental safety. In general, this is a promising direction in environmental protection, in the rationalization of nature management, and in sustainable development.

The tasks of sustainable ecological and economic development in a number of areas continue to be relevant in many areas [118,119,120,121,122,123]. The concept of sanitary safety and environmental safety [124,125,126,127] is fully applicable to the system of rational nature management even with concomitant waste generation of varying intensity. We believe that the administrative and regulatory powers of environmental management can be significantly expanded. Moreover, this is especially necessary in the Republic of Chile, in other countries of South America, in many regions of Africa, in Russia and in the territories bordering it, in a number of Asian countries, and so on. It is economically useful that the formed environmental management systems include approaches to the rationalization of nature management and in relation to waste management. This is necessary in relation to various economic complexes: agriculture, small and large livestock complexes, processing points for various biological objects, and settlements.

## Figures and Tables

**Table 1:** Physicochemical characterization of canine manure, based on Nch 2880. from 2004: *Compost—Classification and Requirements*.

Sample Identification			Canine Manure	Acceptance Level		
Chemical Analysis				Class A	Class B	Method
pH			5.8	5.0	8.5	TMECC 04.11
Electrical conductance		dS/m	9.4	<3	<8	TMECC 04.10
Organic Matter		%	71.0	>20		TMECC 05.07-A
Organic Carbon		%	39.4	>11		TMECC 05.07-A
Total Nitrogen	(N)	%	3.17	>0.5		TMECC 04.02-D
C/N ratio			12.4	<25	<30	TMECC 05.07-A
Available Ammonium	(NH_4_)	mg/kg	1330			
Available Nitrate	(NO_3_)	mg/kg	539			
NH4/NO3 ratio			2.5	<3		TMECC 04.02 -BC
Total Phosphorus	(P_2_O_5_)	%	8.7			
Total Potassium	(K_2_O)	%	0.43			
Total Sulfur	(S)	%	0.22			
Total Sodium	(Na)	%	0.54			
Total Molybdenum	(Mo)	mg/kg	<0.01			
Total Iron	(Fe)	mg/kg	2508			
Total Manganese	(Mn)	mg/kg	446			
Total Boron	(B)	mg/kg	35			
Total Arsenic	(As)	mg/kg	5.30	15	20	TMECC 04.06
Total Cadmium	(Cd)	mg/kg	<0.01	2	8	TMECC 04.06
Total Copper	(Cu)	mg/kg	70	100	1000	TMECC 04.06
Total Chromium	(Cr)	mg/kg	8.7	120	600	TMECC 04.06
Total Mercury	(Hg)	mg/kg	0.950	1	4	TMECC 04.06
Total Lead	(Pb)	mg/kg	11.1	100	300	TMECC 04.06
Total Selenium	(Se)	mg/kg	<0.01			
Total Zinc	(Zn)	mg/kg	878	200	200	TMECC 04.06
Total Sulfur	(S)	mg/kg	640			
Humidity		%	77	30–45		TMECC 03.09
Dry Matter		%	23	70–55		

**Table 2:** Biodigester parameters.

Parameter	Units	Value
Average Annual Temperature	°C	14.2
Dry Matter	kg	6.9
Volatile Mass	kg	4.899
Dilution	L/day	40
Specific Volume	L	66
TRH	Days	30
Solid Retention Time (SRT)	Days	30
Charge Tank	L	82
Discharge Tank	L	272
VOC	Kg MV/m^3^/day	4.9/2.5 = 1.96

## Data Availability

The datasets generated and analyzed during the current study are available from the corresponding author on reasonable request.
